# Captivating Perplexities of *Spinareovirinae* 5′ RNA Caps

**DOI:** 10.3390/v13020294

**Published:** 2021-02-13

**Authors:** Justine Kniert, Qi Feng Lin, Maya Shmulevitz

**Affiliations:** Department of Medical Microbiology & Immunology, Li Ka Shing Institute of Virology, University of Alberta, Edmonton, AB T6G 2E1, Canada; kniert@ualberta.ca (J.K.); qifeng@ualberta.ca (Q.F.L.)

**Keywords:** *Spinareovirinae*, reovirus, capping, RNA, *Orthoreovirus*, *Aquareovirus*, *Cypovirus*, transcription, nucleotide, virus

## Abstract

RNAs with methylated cap structures are present throughout multiple domains of life. Given that cap structures play a myriad of important roles beyond translation, such as stability and immune recognition, it is not surprising that viruses have adopted RNA capping processes for their own benefit throughout co-evolution with their hosts. In fact, that RNAs are capped was first discovered in a member of the *Spinareovirinae* family, *Cypovirus*, before these findings were translated to other domains of life. This review revisits long-past knowledge and recent studies on RNA capping among members of *Spinareovirinae* to help elucidate the perplex processes of RNA capping and functions of RNA cap structures during *Spinareovirinae* infection. The review brings to light the many uncertainties that remain about the precise capping status, enzymes that facilitate specific steps of capping, and the functions of RNA caps during *Spinareovirinae* replication.

## 1. Introduction

Although this review centers on RNA capping by members of the *Spinareovirinae* subfamily, we will first provide a brief introduction to the virus family phylogeny, basic structure, and replication cycle.

**Figure 1:**
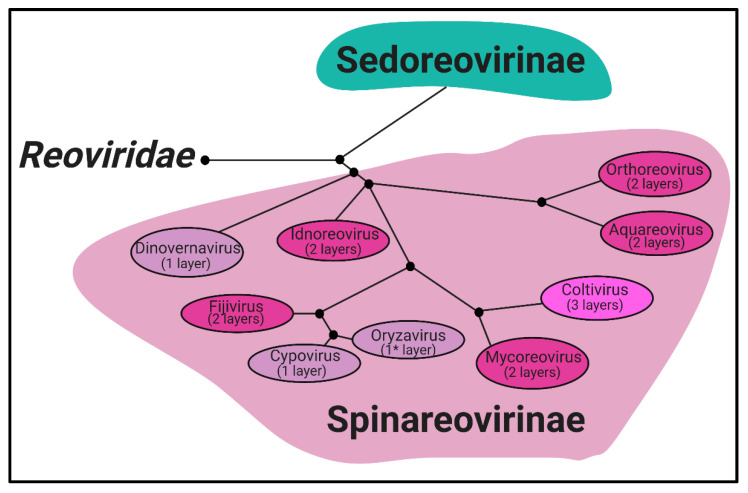
*Spinareovirinae* phylogenetic tree. A phylogenetic tree (neighbour joining) of the *Reoviridae* subfamily, *Spinareovirinae*. Clustering is determined by sequence homology between the RNA-dependent RNA polymerase protein sequences. Capsid layers found among each genus are indicated. *Oryzavirus contains a complete core particle, and a partial outercapsid more closely resembling intermediate subviral particles (ISVPs) seen with *Mammalian Orthoreovirus* (MRV), Figure 2. Figure generated using BLOSUM62 and re-illustrated using Biorender.com. Accession numbers: (AKG65873, ALK02203 for *Orthoreovirus*), (AGR34045, AAM92745, AAL31497 for *Aquareovirus*), (AAM18342, 690891, AAK00595 for *Coltivirus*), (001936004, BAC98431 for *Mycoreovirus*), (AEC32904, AAC36456 for *Oryzavirus*), (AAN46860, AAK73087 for *Cypovirus*), (Q8JYK1, BAA08542 for *Fijivirus*), (ABB17205 for *Idnoreovirus*) and (AAZ94068 for *Dinovernavirus*).

Phylogeny. *Reoviridae* is a family of segmented, dsRNA viruses divided into two subfamilies based on the presence of “turrets/spikes” at the vertices of the viral capsid [1]. The non-turreted members of the *Reoviridae* family form the *Sedoreovirinae* subfamily, while the turreted viruses form the *Spinareovirinae* subfamily, the focus of this review. The *Spinareovirinae* subfamily is composed of nine genera: *Orthoreovirus*, *Aquareovirus*, *Coltivirus*, *Mycoreovirus*, *Cypovirus*, *Fijivirus*, *Dinovernavirus*, *Idnoreovirus*, and *Oryzavirus*, with a wide host range including vertebrates, invertebrates, plants, and fungi. Phylogenetic trees based on the amino acid (aa) sequences of the RNA-dependent RNA polymerases (RdRps) have been built to visualize the relationship between each virus, as depicted in Figure 1. Though structurally conserved, when comparing aa sequences of homologous proteins among the genera, there is often less than 26% sequence similarity. However, protein regions with structural and/or enzymatic function are typically more similar than the rest of the protein [1].

**Figure 2:**
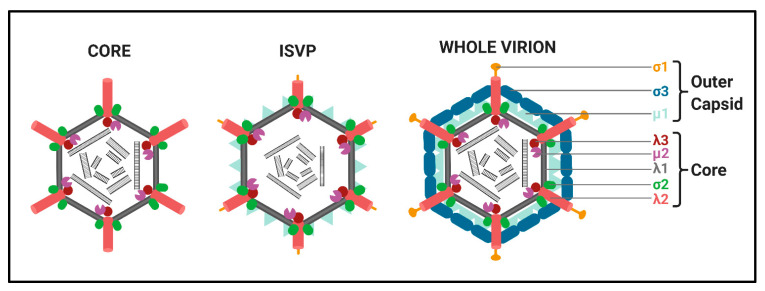
Structures of *Mammalian Orthoreovirus (MRV) 3.* **Left**: Transcriptionally active MRV cores composed of the λ1 shell (grey) held together by the σ2 clamps (green). The λ2 turrets (red) are found at each vertex, below which the RNA-dependent RNA polymerase (RdRp) λ3 (maroon) and μ2 (purple) are predicted to sit; **Middle**: Intermediate subviral particles (ISVPs) are generated through proteolytical cleavage within endosomes after cell-entry. The σ3 outercapsid protein is completely cleaved away, while μ1 (light blue) is cleaved to δ. The σ1 attachment protein (yellow) is also cleaved at the N-terminus. ISVPs may also be generated in the natural niche, which for MRV is the intestinal tract; **Right**: Transcriptionally inactive whole virions are generated from core particles becoming decorated with σ3 (dark blue) and μ1 heterohexamers, forming the outercapsid. Additionally, complete σ1 proteins also attach to the λ2 pentamers. Figure generated using Biorender.com.

Structure. All *Spinareovirinae* members share the same, basic virion structure: concentric layers of proteins with icosahedral symmetry encapsidating the segmented dsRNA genomes (Figure 2) [1]. The number of layers varies among the genera, as depicted in Figure 1. All *Spinareovirinae* members consist of a core, or innermost particle, which is transcriptionally active with T = 1 symmetry. The core particle is formed by 60 asymmetric homodimers of the major-core structural protein, λ1 of Mammalian *Orthoreovirus* (MRV, or “reovirus” henceforth) [2,3]. All proteins discussed hereafter will be given the MRV designation; however, it is important to recognize that homologous proteins of the other genera may have different names. Enforcing this backbone structure are the “clamp” σ2 proteins, which are only present among *Spinareovirinae* [2,4]. MRV contains 150 copies of the clamp protein, distributed about the 5-, 3-, and 2-fold axes [2]. At the twelve 5-fold axes sits the pentameric “turrets”, formed by the λ2 protein [2,5]. These turrets form hollow cylinders and possess both methyl- and guanylyl-transferase activity (MTase and GTase, respectively), discussed in detail in later sections. Among all the genera, there are always 12 turrets; however, their shapes and configurations vary. For example, *Oryzavirus* has both wider and taller turrets compared to MRV [4]. The shape of the λ2 turrets may reflect differences in infection and transcriptional mechanisms; when the outercapsid of MRV is cleaved away, the λ2 turrets take on a more “open” conformation, potentially facilitating the release of mRNA into the host cytoplasm [6].

Some *Spinareovirinae* members, such as *Oryzavirus*, *Cypovirus*, and *Dinovernavirus*, do not have additional layers beyond the core particle. In contrast, *Orthoreovirus*, *Aquareovirus*, *Coltivirus*, *Mycoreovirus*, *Fijivirus*, and *Idnoreovirus* have layers of outercapsid proteins with considerable structural similarity that surround the innermost core particle, forming a T = 13 symmetrical structure. (Figure 2) [1]. Two major outercapsid proteins, μ1 and σ3, form heterohexamers (200 units for MRV) that attach onto the core particles, forming the T = 13 lattice [3,7]. The μ1 proteins found within these complexes contact both the σ2 clamps and λ2 turrets to anchor the outercapsid onto the core [2,3]. In the case of MRV, there is an additional outercapsid receptor binding protein that is absent among the other *Spinareovirinae* genera, σ1, known to bind both junctional adhesion molecule A (JAM-A) and sialic acids (SAs) on the surface of mammalian cells [1,8].

Members of the *Spinareovirinae* subfamily have genomes consisting of 9–12 dsRNA segments, depending on the genera [1]. Most of these dsRNA segments encode a single viral protein, thus they are largely monocistronic. *Spinareovirinaes*’ genome segments share conserved sequences at their termini with 3′ terminal regions (UCAUC-3′ for MRV) typically more conserved than the 5′ sequences [1]. This may suggest functions for the non-coding regions of reovirus genomes, potentially in the regulation of transcription or encapsidation. As will be discussed in detail throughout this review, the (+)-sense RNAs of *Spinareovirinae* are capped. Unlike eukaryotic mRNAs, both genomic and transcribed *Spinareovirinaes* RNAs lack 3′-polyA tails.

**Figure 3:**
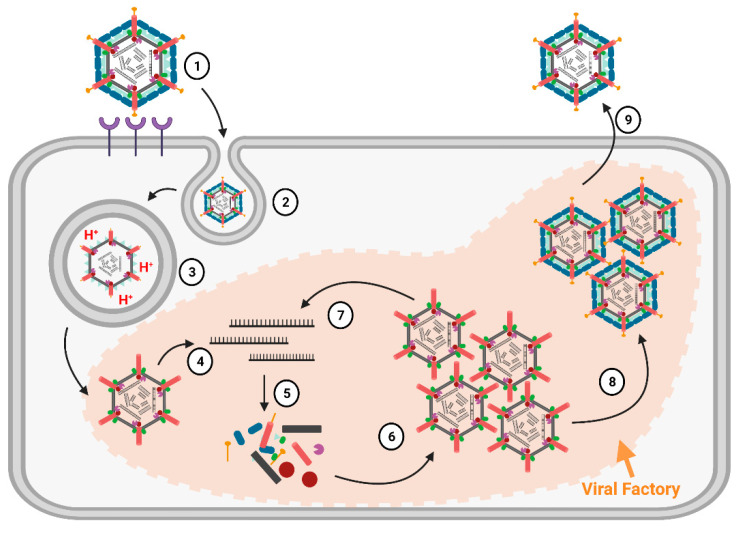
Replication cycle of *Mammalian Orthoreovirus 3*. (1) Receptor-mediated attachment of complete virions via σ1 to junctional adhesion molecule A (JAM-A) and/or sialic acid (SA) residues on the mammalian cell surface; (2) Following membrane attachment, virions enter the cell via clathrin-mediated endocytosis; (3) Endosomal acid-dependent proteases digest the MRV outercapsid, generating ISVPs before the eventual release of core particles into the cytoplasm; (4) Now-transcriptionally active core particles produce mRNAs (initial replication) using the viral λ3 polymerase; (5) After being secreted through the λ2 channels, mRNAs are translated into proteins. Viral factories are formed which serve as sites for viral replication, and (6) assemble into more core particles; (7) These newly generated cores then transcribe more mRNA, which in turn is translated into more proteins and assembles into more cores; (8) Outercapsid proteins assemble onto cores, halting transcription and producing progeny fully-infectious virions; (9) Mature infectious virions egress from the cell and disseminate. Figure generated using Biorender.com.

Replication Cycle. We will provide a brief overview of the replication cycle for MRV here, as illustrated in Figure 3; however, readers are directed to a recently published review [9] for a more in-depth description. Infection begins with entry of the virus into host cells, a process mediated by the attachment of the σ1 receptor binding protein to sialic acids (SAs) and/or junctional adhesion molecule A (JAM-A) residues [10]. In cell culture, MRVs are generally internalized by clathrin-mediated endocytosis, but can also internalize by phagocytosis or pinocytosis [11]. Within endosomes, acid-dependent proteolytic processing allows stepwise degradation of the outercapsid, beginning with σ3 degradation followed by μ1C cleavage, generating intermediate subviral particles (ISVPs) (Figure 2) [12]. Eventually, transcriptionally active core particles penetrate into the cytoplasm of the cell [12]. Given the natural niche for MRV is the intestine, it is believed that intestinal enzymes such as chymotrypsin kickstart the proteolytic degradation required for infection, generating ISVPs that can penetrate host membranes directly [13]. While MRV entry is well characterized, modes of entry for other *Spinareovirinae* members likely vary. For example, some *Orthoreoviruses* express a membrane fusion-inducing protein and can make use of syncytium formation for cell-to-cell spread [14].

An important feature of *Spinareovirinae* is that RNA transcription occurs within the core particles, and full-length mRNAs are extruded from the λ2 pentameric turrets. As will be comprehensively discussed in this review, the RNAs can also be capped before extrusion to the cytoplasm. The (+)-sense RNAs serve as messenger RNAs for the expression of viral proteins by the host translation machinery. Moreover, as de novo viral capsid proteins are synthesized, (+)-sense RNAs can also be encapsidated into progeny cores. Within progeny cores, (-)-sense RNAs are transcribed, producing the dsRNA genome that serves to amplify (+)-sense RNA synthesis. Primary transcription refers to the first RNA molecules produced by the incoming core particles, while secondary transcription refers to RNA synthesis by newly assembled core particles (Figure 3). The replication of reovirus occurs within localized areas called factories, which are composed of both viral proteins (μ2, μNS, and σNS) and host materials (endoplasmic reticulum (ER) fragments, microtubules, and possibly more) [15]. Ultimately, whole progeny viruses are assembled that can egress from cells, predominantly in a non-lytic manner [9].

This review focuses on RNA capping in the context of the *Spinareovirinae* infection cycle, summarizing what is known but also revealing the abundant questions that remain. The review first explores what we know conclusively about the structure of 5′caps found on reovirus (+)RNAs; this remains a debated question despite decades of research. Secondly, the review examines what is known about the role(s) of RNA caps during virus replication; while 5′caps are well recognized to promote protein translation, recent studies suggest that RNA caps might play additional roles in the reovirus life cycle. Lastly, this review asks how reovirus RNAs obtain their cap in the first place? While the capping reaction is well understood, attributing each enzymatic step to a specific virus protein has proven to be challenging. Though seemingly a simple guanosine with a reverse 5′ to 5′ triphosphate linkage, many perplexities remain about the 5′ caps of *Spinareovirinae*.

## 2. Capping Status of Viral RNAS

**Figure 4:**
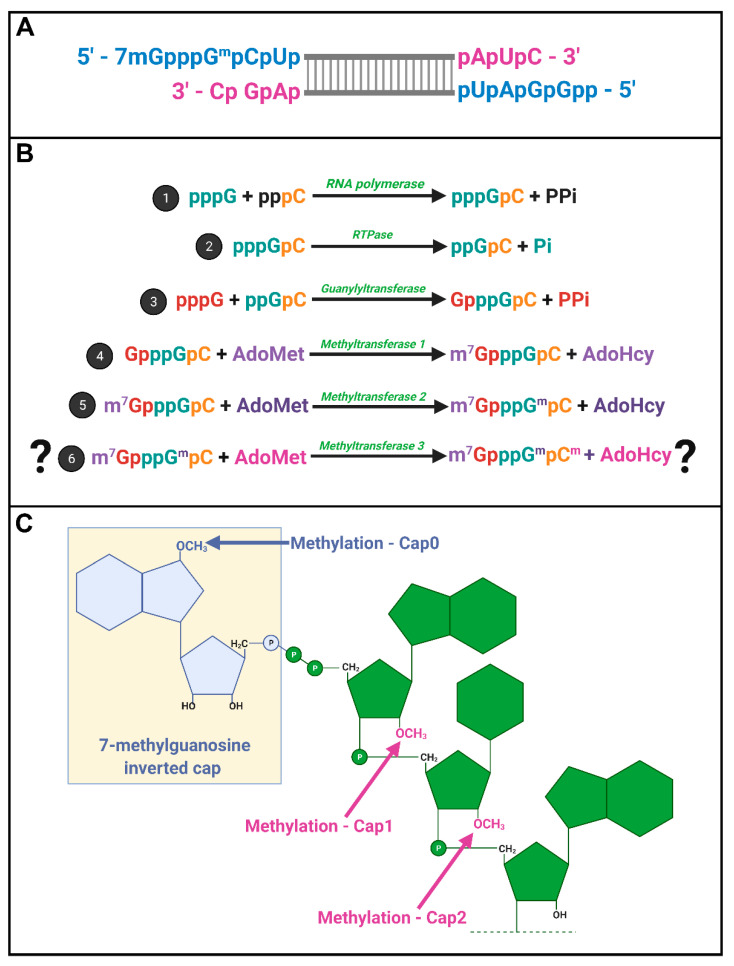
mRNA capping by *Spinareovirinae*. (**A**) A depiction of the cap structures on 5′ termini of a dsRNA segment; (**B**) Proposed stepwise enzymatic generation of cap structures found within *Spinareovirinae* RNA. An RdRp catalyzes the first step of the reaction, production of (+)RNA. Secondly, RTPase-mediated hydrolysis of the γ phosphate produces diphosphate RNA 5′ ends. In the third step, guanylyltransferase activity mediates the addition of guanosine via a reverse 5′ to 5′ triphosphate linkage. Methyltransferases then mediate methylation at the N7 position of the guanosine cap (step 4), and at the 2′O position of the adjacent first mRNA nucleotide (step 5), producing cap0 and cap1 structures sequentially. While it is debatable whether reovirus RNAs have additional methylation of the second RNA nucleotide to produce cap2 structures, such an activity would also require a methyltransferase; (**C**) Depictions of the molecular structures of RNA cap0, cap1, and cap2 with key methylated residues. Figure generated using Biorender.com.

*Spinareovirinae* +RNAs have Cap1 structures. The years 1974–1976 were riveting for the topic of mRNA capping; Shatkin, Lengyel, and Kozak, along with their trainees and colleagues, discovered that mRNAs were capped and unravelled the enzymatic steps involved in cap addition and modification (Figure 4) [16,17,18,19,20,21,22,23,24,25,26,27]. We recommend visiting these original publications to appreciate how this pivotal discovery was made and disseminated using radioisotopes such as ^3^H and ^32^P, enzymatic or chemical treatments, chromatography, brilliant experimental design, and a typewriter. Miura et al. (1974) first discovered that the 5′-terminal phosphates of MRV-synthesized RNAs were in a blocked configuration and could only be labelled by [^32^P] if the RNAs first underwent oxidation, beta-elimination, and phosphomonesterase treatment [28]. Upon removal of the blocking group, they found that the first nucleotide was always a modified guanine, thought probably to contain a 2′-*O*-methyl group, followed by a cytosine (GpCp). It is now well recognized that a 5′-GCUA sequence is indeed common to all ten MRV genome segments.

Motivated by the finding that RNAs of several different viruses, including MRV, also contained methylated nucleotides [29,30,31,32], Shatkin’s group set out to define the precise composition of the 5′ termini. Through a series of differential enzymatic treatments and interpretation of chromatographic peaks, the Shatkin group showed that MRV cores synthesize mRNAs with a guanylate cap added to the 5′ end through a 5′-5′ inverted linkage (G(5′)ppp(5′)GpCp), rather than the conventional 5′-3′ linkage. The Shatkin lab also discovered that methyltransferase activities add a methyl group from S-adenosyl-methionine (SAM) to the N7 position of the guanosine cap and the 2′O position of the adjacent first mRNA nucleotide (which is a guanine for MRV) to produce m7G(5′)ppp(5′)Gmp; a structure later termed “cap1”. It was already known that a viral-associated RNA triphosphatase (RTPase) could remove the γ-phosphate of nucleotides [33,34]; this hydrolase activity was now predicted to produce the diphosphate 5′guanine-cytosine terminus (5′ppGpCp) to which the inverted guanosine cap is added. One month following their description of the cap1 structure on in vitro synthesized RNAs, the Shatkin team demonstrated that the same cap1 structure was found on RNA genomes of viruses purified from infected cells; this suggested that cap1 structures are indeed produced during MRV infection and not just an artifact of in vitro systems [25]. By 1976, the cap1 structure was observed in numerous viruses and eukaryotes ranging from humans to silkworms to yeast; mRNAs being capped became an accepted dogma. The *Spinareovirinae* genomic dsRNA was then found to consist of capped (+)-sense RNAs, but diphosphate bearing (−)-sense RNAs that are uncapped and unmethylated at the 5′ end (Figure 4A).

In 1976, Furuichi et al. depicted the sequence of capping reactions along with the substrates and enzymatic activities involved [16,17,20]. Specifically, RTPase, guanylyltransferase (GTase), and two methyltransferases (MTases) are necessary, in that order, to generate the final cap1 structure on reovirus RNAs (Figure 4B). Later sections will discuss the possible location and orchestration of these enzymes in the context of *Spinareovirinae*.

Does reovirus also make cap2 structures? In 1976, Desrosiers et al. found that in L929 cells, 50% of MRV derived mRNAs also had a methyl group on the 2nd nucleotide not counting the guanosine cap; a structure referred to as cap2 (m7G5′ppp5′GmpCmp) (Figure 4C) [35]. The presence of cap2 structures on reovirus RNAs was also suggested by Shatkin and Both [19]. Specifically, while (+)-sense RNAs generated in vitro by reovirus cores had cap1, ~40% of (+)-sense RNAs synthesized during the infection of L929 cells at 5–11 h post infection (hpi) had cap2 structures. Moreover, cap2 was absent from the (+) strand of the dsRNA genome, suggesting that packaged (+)RNA was cap1-modified while ~40% of unpackaged (+)RNAs had cap2 structures [36]. Based on these findings, the authors proposed that cap1 versus cap2 structures may help regulate the fate of (+)RNAs, distinguishing protein translation versus packaging.

An interesting twist to the possibility of cap2 structures on reoviral RNAs is that interferon (IFN) signaling was found to lower reovirus cap2-associated methylation. Specifically, during in vitro reovirus core transcription reactions, the addition of cell lysates from IFN-treated Ehrilich ascites tumor cells impaired cap2-associated methylation, relative to cell lysates from untreated cells [27,37]. During bonafide infection of L929 cells, 36–47% fewer cap2-modified viral RNAs were detected following IFN treatment [36]. These early studies beget many unresolved questions. First, are reovirus (+)RNAs indeed differentially methylated; for example, can these findings be reproduced by other laboratories and can cap2 structures be identified in additional cell types during reovirus infection? Given that over 30 years have passed since the original discovery of cap2 on reovirus RNAs, it is important that these findings be validated with recent technologies in different experimental systems. If reovirus RNAs indeed have cap2 structures, then are cellular or viral enzymes responsible for methylating the 2nd nucleotide? Do the methylations affect the fate of viral RNAs, for example, deciphering between packaging into progeny particles versus becoming templates for protein translation? Given that RNA capping and methylation impact the detection of foreign RNAs as “non-self”, what role does cap1 play in thwarting the activation of cellular antiviral signaling in response to reovirus, and might cap2 also play a role in subverting host responses? Reciprocally, do cells respond to antiviral signaling by modifying the methylation of RNAs as a strategy to reduce virus replication? Box 1 highlights a few of the current unanswered questions regarding reovirus cap2 structures.

Box 1 **Unanswered Questions—Cap2 Structures.**

Do unpackaged reovirus (+)RNAs have cap2 structures? If so…

- Is cap2 methylation mediated by cellular or viral enzymes?
- What is the function of cap2? Is it involved in packaging and/or translation?
- What effects do cap1 and/or cap2 structures have on host signalling and antiviral responses?
- Is cap2 common to multiple cell types permissive to reovirus?

In vitro evidence of uncapped RNAs: A very interesting possibility proposed in 1980 was that reoviruses also produce uncapped RNAs; but similar to cap2 structures, the interpretations would benefit from further experimental examination. In Zarbl et al. (1980) and Skup and Millward (1980), the authors fractionated virions from reovirus infected L929 cells on a glycerol gradient, and progeny subviral particles (SVPs) were categorized as the slow-sedimenting regions of the gradient relative to mature virions and parental SVPs [38,39]. The fractions were then subjected to in vitro transcription assays in the presence of ribonucleotides. SAM was either added or omitted to modulate capping. In vitro synthesized RNAs were then extracted, precipitated, and further purified by sucrose density gradient. The fast-sedimenting (mature) virions did not incorporate [^3^H] cytidine monophosphate (CMP) or [methyl-3′H]SAM unless treated with chymotrypsin; this is expected given that mature virions must uncoat to cores to become transcriptionally active. What was surprising, was that the slow-sedimenting virions (“progeny SVPs”) incorporated [^3^H]CMP but not [methyl-3′H]SAM, unless also treated with chymotrypsin. From these findings, they suggested that later during infection, progeny produce uncapped mRNAs. However, a close look at the composition of the fast (mature) and slow (“progeny SVPs”) virions generates some uncertainty with respect to what the experiments actually reflect. The authors describe that for both fractions “the relative intensity of staining in the λ1 and σ1 regions was similar”, but that “polypeptides µ1, µ2 and σ3 were clearly absent in the SVPs found in the slow-sedimenting fractions”. These findings raise some confusion as to what the slow-sedimenting particles actually represent, given that progeny cores that would amplify RNA synthesis in secondary transcription (Figure 2) are not expected to have absence of µ2 and presence of σ1 as described for the “progeny SVPs”. Are these assembly intermediates rather than progeny cores? Could the process of purification produce particles that do not fully recapitulate viral structures and activities found within the cell? Do the activities of purified fractions in an in vitro transcription reaction recapitulate processes found during natural infection? Now that we have more sensitive assays, such as mass spectrometry (MS), nuclear magnetic resonance (NMR), and cryo-electron microscopy (cryo-EM), to characterize protein composition, and even 3D structures of reovirions, it would be interesting to revisit these “progeny SVPs” and decipher their composition and structure.

In cellulo evidence for uncapped RNAs: The most convincing evidence for the production of uncapped RNAs during reovirus amplification came in 1981 by Skup, Zarbl and Millward [40]. Here, the authors added [^32^P]orthophosphoric acid during reovirus infection at either early (3.5–6.5 hpi), intermediate (6.5–9.5 hpi) or late (10–13 hpi) time points, then isolated polyribosomal RNAs by high salt precipitation and sucrose gradient sedimentation, purified reovirus RNAs by hybridization to genomic RNA, and assessed 5′terminal structures. Their findings suggested a switch from m7G(5′)ppp(5′)GmpCp (cap1) to pGp 5′-termini as infection progressed, with only 2% of ribosomal reovirus mRNAs containing cap1 at the 10–13 hpi time point. Given the potential significance of these findings, it would be valuable to reproduce and validate the enrichment of uncapped RNAs with modern methods. For example, it would be important to include an assessment of the purity and identity of fractions dubbed “polyribosomal” and ensure that fractionation methods accurately represent the majority of ribosomal mRNAs. For instance, given recent discoveries that the reovirus-translating ribosomes segregate in virus factories, did the methods by Skup, Zarbl and Millward also capture these species? Furthermore, given the recognition that reoviruses vary genetically and phenotypically from each other, even among different laboratory strains of serotype 3 Dearing (T3D) MRV, it would be interesting to determine whether uncapped RNAs are common to a broader collection of reovirus isolates [41,42,43]. Similarly, is this a unique characteristic of L929 cells or can it be observed in other cell types permissive to reovirus infection? It is also curious as to why cap2 structures were observed by Lengyl’s group but not by the Millward group. The authors assessed only polyribosome fractions, but it would be interesting to quantify the proportion of total viral RNAs that are uncapped versus capped; this being important for determining if there is an enrichment of uncapped RNAs in ribosomes.

If uncapped RNAs can be confirmed, then many questions remain about the possible role and implications of uncapped RNAs. As described below, the cap structure was recently proposed to serve as a beacon for RNA encapsidation, so how would uncapped RNAs affect assembly? Finally, what function would pGp 5′ ends serve in the reovirus replication cycle? In other words, what could be the functional relevance behind the production of uncapped mRNA at later stages of infection? Moreover, what determines the switch to uncapped RNAs; are substrates limiting or do enzymatic activities and perhaps core conformations change? In addition to L929 cells, can uncapped RNAs be generated in other cell types, and importantly, are uncapped RNAs beneficial to viruses or rather a consequence of host response that is unfavorable to reovirus? Unfortunately, to our knowledge, the findings of uncapped RNAs have not been revisited since 1981 despite their potential importance. Unanswered questions regarding *in cellulo* reovirus capping are highlighted in Box 2.

Box 2 **Unanswered Questions—*In cellulo* capping.**

(1) Is reovirus RNA capping cell-type dependent?
(2) Are reovirus RNAs uncapped at later times in infection? If so…
  - What is the function of uncapped RNAs?
  - Are uncapped RNAs common to all reovirus isolates?
  - Do uncapped RNAs affect antiviral signaling, and/or vice versa?

## 3. Functions of RNA Capping during Infection

Viral RNA transcription is not contingent on capping activity. As early as 1975 when the 5′cap structure was being discovered, there were speculations about its possible functions. Early studies demonstrated that capping was not essential for viral RNA transcription. Specifically, Reeve et al. [44] showed that the addition of [γ-S]GTP effectively prevented capping by resisting hydrolysis to the diphosphate (ppGpCp) substrate required for the capping reaction. Importantly, the addition of [γ-S]GTP did not interfere with viral RNA synthesis in vitro [44]. It would be interesting to perform similar studies in cell culture infections, not only to confirm that capping is dispensable for viral RNA synthesis, but as a strategy to explore the role of capping during reovirus replication. Noteworthy is that although [γ-S]GTP did not prevent RNA transcription, this does not indicate whether the RNA template within the virus particle had to be capped to serve as a template, as the addition of [γ-S]GTP does not affect the capping status of the input genomic template.

RNA capping promotes virus protein translation and RNA assembly. Unlike for viral RNA synthesis, the importance of mRNA capping for protein translation was well established by the 1980s. MRV mRNAs produced in the presence of methyl-donor SAM exhibited heightened ribosome binding and efficient translation in wheat germ or L929 cell lysate-based cell free translation systems [21,45]. These early studies inferred that 5′caps are involved in ribosome recruitment. Marilyn Kozak and Aaron Shatkin then demonstrated in 1976 that, indeed, the 43S ribosome initiation complex was recruited to the 5′terminus of mRNAs [18]. A higher proportion of 43S-protected fragments was obtained using methylated versus unmethylated mRNA, suggesting that m7G contributes to increased efficiency of 43S binding but is not absolutely required [45]. Furthermore, the 80S ribosomal complex was suggested to be formed at AUG-containing regions by recruitment of the 60S ribosomal subunit, since cap-containing RNAs without AUG start codons showed neither 43S nor 80S ribosome rebinding.

Given that mRNA capping promotes translation by recruiting the 43S ribosome complex to the initiation AUG site, the next logical question to ask would be: to what extent does the capping of reovirus mRNA enhance protein translation and assist in competition for host translation machinery? To begin addressing this question, our lab recently used the reovirus reverse genetics approach to compare de novo virus protein synthesis and progeny production in the presence versus absence of mRNA capping [46]. Specifically, transcription of all ten plasmid-derived MRV genome segments was driven by a T7 RNA polymerase promoter as is typical for the reverse genetics system, but the baby hamster kidney (BHK) cells were also variably transfected with the African Swine Fever Virus NP868R capping enzyme. In these experiments, it is important to point out that host capping enzymes are normally found in the nucleus where they remain inaccessible to reovirus particles present in the cytoplasm, thus the transfection of an exogenous capping enzyme allows mRNA produced in the cytoplasm to be capped. With that in mind, the inclusion of NP868R increased reovirus protein levels by ~10-fold, yet increased new progeny particle production by ~100-fold. These experiments suggested that mRNA capping serves additional functions beyond protein expression. To determine if capping promotes translation-independent steps of reovirus replication, BHK cells were infected with reovirus to produce capped viral RNAs, but also transfected concurrently with a plasmid-derived S1 genome segment modified to encode the green fluorescent protein, UnaG. Importantly, the experiments were conducted in the presence or absence of NP868R to produce capped or uncapped S1-UnaG mRNAs, respectively. In this experimental system, the virus infection produced all components for virus amplification and assembly, and the fate of capped or uncapped S1-UnaG alongside the infection could be monitored. Purified virions from the infected-transfected BHK cells were then added to Ras-transformed NIH3T3 cells, and the expression of UnaG from progeny virions was assessed by RT-qPCR and flow cytometry. S1-UnaG expression was only observed when progeny virions were produced in the presence of NP868R. These findings can be interpreted in two ways: (1) capping was essential for S1-UnaG mRNA to be encapsidated, or (2) both capped and uncapped S1-UnaG mRNAs were encapsidated but capping was essential for the subsequent transcription and expression of S1-UnaG transcripts. As discussed below, the reovirus polymerase has a cap-binding site that was previously suggested to serve as an anchor for the viral genomic RNA during transcription. Thus, a feasible possibility to explain the lack of infectious progeny with uncapped S1-UnaG is that the association of the polymerase with RNA caps is essential for RNA encapsidation, transcription, or both.

Aside from its roles in virus replication, the composition of the 5′ termini of RNAs can affect the host response to virus infection. It is now well established that viral RNAs are recognized as foreign by pattern recognition receptors such as toll-like receptors (TLR), RIG-I–like receptors (RIG-I), double-stranded RNA-activated protein kinase (PKR), and melanoma differentiation–associated protein 5 (MDA5). The binding of viral RNA to these receptors results in type I IFN induction and downstream expression of IFN-stimulated genes (ISG), many of which have antiviral properties. RIG-I was found to be the key pattern recognition receptor during MRV infection [47], as shRNA-mediated silencing of RIG-I prevented IFN production following reovirus infection. Additionally, while Ras-transformed cells with impaired RIG-I signaling permitted efficient reovirus dissemination, the silencing of RIG-I in non-transformed cells with functional RIG-I-signaling was necessary to allow efficient MRV cell-to-cell spread. In these studies, either MRV infection or transfection of in vitro synthesized m7G-capped but unmethylated reovirus (+)RNAs induced IFN-dependent antiviral effects. It would be interesting to repeat these studies with differentially modified reovirus (+)RNAs, cap1 or cap2 structures, to determine the role of methylation for RIG-I detection. Another study showed that 5′diphosphate-bearing reovirus -RNAs were also able to induce the IFN response in a RIG-I-dependent manner [48]. In these experiments, total RNA isolated from reovirus-infected cells, genomic RNAs extracted from reovirions, and in vitro synthesized 5′ diphosphate-bearing RNA fragments activated IFN signaling; these effects were reduced by pre-treatment of RNAs using calf intestinal phosphatase, implicating an important role for the 5′ phosphates present on the RNAs in triggering the IFN response. Given that reoviruses evolved to conceal their genomic RNAs in core particles as well as virus factories, it would be interesting to see whether sufficient genomic 5′diphosphate-bearing (-)RNAs “leak” during infection to become primary activators of RIG-I, for example, in the context of unstable capsids. Alternatively, is the activation of RIG-I during a natural reovirus infection primarily due to aberrantly capped or methylated (+)RNAs, or is it caused by additional features in reovirus RNAs that have yet to be characterized? Finally, as mentioned above, if it is confirmed that MRV produces uncapped RNAs with pGp 5′ ends late during infection, how well are such ends recognized by RIG-I and would pGp 5′ ends provide an advantage or disadvantage for the virus with respect to innate immune activation? Unanswered questions regarding the function of reovirus RNA caps are grouped in Box 3.

Box 3 **Unanswered Questions—Function of RNA caps.**

What is the contribution of 5′ diphosphates on genomic (-)RNA and uncapped, cap1, and cap2 structures on genomic and progeny (+)RNAs to…

- RNA assembly?
- RNA transcription?
- Antiviral signaling?

## 4. Capping Enzymes—Structure and Function

**Step 1: RNA-dependent RNA polymerase catalyzes transcription.**

**Figure 5:**
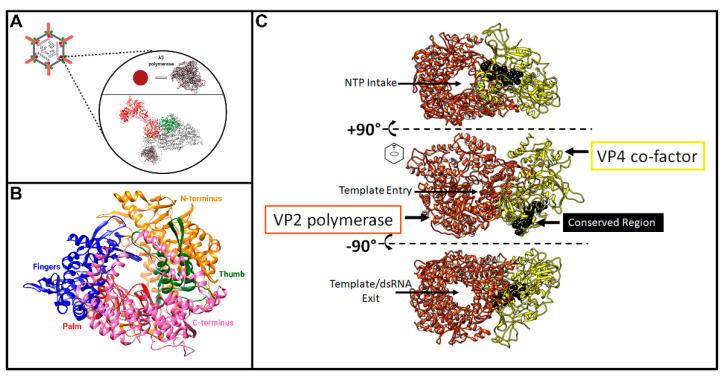
Structure of the *Spinareovirinae* RNA-dependent RNA polymerase. (**A**) The MRV λ3 RdRp (pink) (PDB accession #1MUK) depicted in approximate location relative to a single capsid unit (λ2 (red), σ2 (green), and λ1 (grey)) (PDB accession #6XF8); (**B**) Structure of the MRV λ3 protein with each domain denoted by a different colour: N-terminus in orange, Thumb in green, Palm in red, Fingers in blue, and C-terminus in pink (PDB accession #1MUK); (**C**) Structure of grass carp reovirus (GCV) VP2 (λ3 homolog) in complex with VP4 (μ2 homolog) (PDB accession #6M99). Black spheres on VP4 represent the conserved NTPase motifs. Structure was rotated in order to visualize the different channels within the λ3 complex. Figure generated using Biorender.com.

As depicted in Figure 3, step #1, the first step towards capping reovirus (+)-sense RNA is the production of such RNA by the viral RNA-dependent RNA polymerase (RdRp). After confirmation that λ3 was indeed the RdRp [49], subsequent studies worked towards characterizing its structural features and mechanisms of action. Within the core particle, structural studies suggest the λ3 polymerase to be sitting just inside of the λ1 shell, beneath the site of λ2 pentameric channels (Figure 5A) [2]. Although twelve pentameric channels exist, only ten are occupied by polymerase units—presumably one for each genome segment. This may be due to steric crowding within the core particle, or a requirement for proper assembly and genome packaging. Interestingly, another member of *Spinareovirinae*, the Fako virus of the *Dinovernavirus* genus, also contains ten polymerase units, but only nine genome segments [50]. Kaelber et al. investigated the arrangement of polymerase units within the Fako virus core particle, which is structurally homologous to the core of MRV, using cryo-EM and synthetic maps, and found that not only do genome-containing virions have ten polymerase units, but empty subviral particles do as well. Their findings suggest that the number of polymerase units within the core does not correlate with the number of genome segments, and may instead represent a structural integrity requirement for assembly. Now, whether these findings extend to other members of *Spinareovirinae* remains to be investigated.

In 2002, Tao et al. resolved the crystal structure of MRV λ3 at 2.5Å [51], including 1256 residues out of a total 1267. The structure was missing a flexible loop encoded between residues 957–964, one residue at the N-terminus, and two residues at the C-terminus. The authors found a fingers-palm-thumb core surrounded by a N- and C-terminal elaboration, creating a cage-like structure (Figure 5B), similar to other viral RdRps such as human immunodeficiency virus-1 (HIV-1), grass carp reovirus (GCV), poliovirus, and phage ϕ6. The palm domain contains a four-stranded, antiparallel β-sheet, supported by three α-helices. The thumb domain also contains a β-strand followed by three α-helices. A hairpin loop located between the first strand and first helix of the thumb interacts with a loop at the top of the “fingers”, forming a ring-shape around the catalytic site of the palm. Residues 1–380 at the N-terminus of λ3 cover one side of the active site, anchoring the continuous surface between the fingers and the thumb domain. At the C-terminus, residues 981–1267 make up the ring shape, said to be similar to the sliding clamp found in some DNA polymerases; though unlike DNA polymerases, the λ3 structure is unlikely to open and close, as suggested by Tao et al. [51]. This “cage” has four channels leading to the active site (Figure 5C, depicted using the λ3 homolog in GCV, called VP2). Two channels are entry paths for the template strand and rNTPs, and two are exit paths for the (−) strand template or dsRNA products, and for the (+) strand transcript. During transcription, it is thought that the (–) strand RNA moves through the RdRp, which remains in place, while the nascent transcript emerges from the polymerase and is then threaded into the λ2 channels [51]. Templates enter the 3′ active site of the polymerase, forming base pairs with bound rNTPs. The ribose base of the template nucleotide than situates itself under residues P530 and I528, causing the downstream template to bend away from the catalytic pocket [51]. Furthermore, Tao et al. described the specificity of λ3 for ribonucleotides in their 2002 publication, in vivid detail regarding specific interactions and residues for RNA synthesis by the λ3 protein. Once nascent RNA molecules are synthesized, they are believed to be capped and methylated through the λ2 pentameric channels before exiting into the cytoplasm [51].

During nascent strand synthesis, it was shown by early publications that RNA polymerases have different regulatory mechanisms with respect to initiation and elongation steps [52]. This is further supported by the fact that only core particles synthesize full-length mRNAs, but intact whole-virions and cores are both able to synthesize abortive transcripts, produced via the initiation step [52]. To investigate this phenomenon in the context of *Spinareovirinae* (MRV), Farsetta et al. partially re-coated cores with outercapsid proteins μ1, σ3, and σ1 and assessed transcriptional activity [53]. They found that the amount of full-length mRNA produced was inversely proportional to the number of μ1-σ3 complexes bound, yet σ1 levels had no effect. It is possible that upon μ1-σ3 binding, the λ2 pentamers narrow and mediate transcriptional shut-off, though this remains to be established experimentally [3].

Interestingly, Tao et al. mapped a 5′cap binding site on the surface of the polymerase “cage”, between the template entrance and exit channels, suggesting a template retention mechanism through which the 5′ end of the (+) sense strand facilitates the insertion of the 3′ end (−) strand into the template channel [51]. To directly investigate if λ3 recognizes the 5′ cap structure, they soaked their λ3 crystals with an analog of m7G(5′)ppp(5′)G mRNA cap, as well as the conserved tetra-nucleotide 5′-GCUA-‘3 sequence [53]. They discovered that the tetrameric sequence did not bind significantly well to λ3, but the cap structure did. The authors further surmised that the cap binding and enzymatic activities of λ3 are independent of each other, since RNA polymerization experiments using uncapped ssRNA template resulted in the production of abortive transcripts 2–4 bases in length despite the absence of a 5′ cap. Two purposes were proposed for the cap binding activity of λ3: (1) to facilitate RNA packaging into the assembling cores, and (2) to anchor the dsRNA genome segment by the 5′ end of (+) sense RNA, so that the 3′ end of the (−)-sense RNA is primed for insertion into the polymerase complex and can serve as a template for mRNA synthesis. These predictions are congruent with our finding described above [46], whereby transfected T7-driven UnaG-expressing S1 segment, alongside infection by MRV, produces 50-fold more infectious UnaG-expressing virions when T7-NP868R capping enzyme was co-transfected. Future experiments should delineate the precise purpose of λ3-cap interactions by distinguishing whether uncapped segments fail to be assembled at all, versus fail to facilitate transcription.

**Step 2: Phosphatases hydrolyze the γ-phosphate from the 5′ end of (+)RNA.**

Once (+)RNA molecules have been synthesized by the viral RdRp, the next step in the capping reaction is cleavage of the 5’-terminal γ-β phosphoanhydride bond (Figure 3, step #2). RNA 5’-triphosphatases (RTPases) are enzymes that cleave this bond, generating 5′ diphosphate-ended RNA and a phosphate anion from 5′ triphosphate-bearing RNA molecules, as depicted in Figure 4. The resulting 5′-diphosphate-end enables the addition of a guanine cap on the 5′ terminus of mRNA. RTPases come in two types: those encoded by viruses, yeast, and protozoan are metal-dependent and possess nucleotide-5′-triphosphatase (NTPase) activities, while mammals, plants and other higher eukaryotes use metal-independent cysteine-type phosphatases. It is important to point out the differences between RTPases and NTPases as they pertain to the capping reaction: RTPases act on polynucleotides, such as RNA molecules, while NTPases act on nucleotide monomers. Furthermore, both activities can be present on the same protein as described for metal dependent RTPases, including those encoded by viruses.

For *Spinareovirinae,* NTPase and RTPase activities have long been investigated, but as the descriptions that follow will unveil, the precise enzyme that fulfils the RTPase step in RNA capping remains to be empirically resolved. NTPase activity was first reported in 1970 by Kapuler et al. (1970) [54], with specific hydrolysis preference of rATP > rCTP = rGTP > rUTP for ribonucleoside triphosphates to diphosphate. Importantly, rates of hydrolysis of particular ribonucleotides by specific NTPase were insensitive to concentrations of other rNTPs; for example, rates of GTPase reactions were insensitive to rUTP and rATP concentrations. Conversely, CTPase and ATPase activities were heat inactivated at the same rate, suggesting that both activities were associated with a single protein. These findings suggested that while some activities lie in a single protein (e.g., CTPase and ATPase), there may also be separate base-specific activities dispersed among distinct proteins, or among distinct vertices of reovirus particles. To our knowledge, the biological relevance of these interesting findings has yet to be resolved. Another interesting question that was posed was what purpose NTPase activities serve, given that such activity would deplete the nucleoside triphosphate substrates needed by the RdRp to synthesize RNAs. Some suggestions included (1) to deplete dNTPs for the host, (2) to hydrolyze the γ-phosphate from RNA chains beginning with pppNp (i.e., RTPase step of capping), (3) to regulate polymerase activity, for example, ceasing transcription activities to promote virion assembly, and/or (4) to provide energy for dsRNA and ssRNA unwinding during transcription and (-)RNA strand synthesis, respectively. To our knowledge, functions (2) and (4) are the ones currently being pursued experimentally, and the question of whether NTPase depletes ribonucleoside triphosphate pools has yet to be explored.

Having identified NTPase activity in reovirus, the immediate next question became which reovirus protein(s) provide such activity. MRV serotype 1 Lang (T1L) and serotype 3 Dearing (T3D) were found to possess distinct NTPase characteristics, such as reaction kinetics at varying pH, temperatures, and cation requirements. By monitoring strain-specific differences in ATPase and GTPase activities between T1L and T3D reassortments, both λ1 [55] and µ2 [56] were discovered to either mediate or modulate NTPase activities. Furthermore, using temperature-sensitive mutants and serotype reassortments, µ2 was associated with RNA synthesis efficiencies during in vitro transcription reactions and cell culture infections [57,58]. While ATPase activities were ascribed via reassortment analysis to λ1, with µ2 as a secondary determinant, GTPase activities were dominantly determined by the strain-specific differences in µ2 [56]. Indirectly, this makes µ2 a better suspect for RTPase activity during capping, since G is the first base of reovirus RNAs. On the other hand, λ1 may fulfill the ATPase-dependent dsRNA binding and helical activities. Since these early studies, λ1 and µ2 have remained the major candidates for NTPase and RTPase activities. However, importantly, for both λ1 and µ2, being strong determinants does not imply a direct role in NTPase/RTPase activities, as these proteins could be indirect modulators for these processes by affecting other protein or core structures.

Lambda 1: MRV λ1 was speculated to provide the RNA helicase activity required to unwind dsRNA templates during transcription, owing to its strong ATPase activity and the presence of sequences resembling the three common motifs of helicases: (1) PRKTKGKS at amino acids 5–12, similar to the ATP binding motif of ATPase A (A/G)xxxxGK(S/T) (also known as Walker A), (2) DEAD at amino acids 100–103, similar to the DEAD or DExH motifs (also known as Walker B) found in RNA and DNA helicases, and (3) NRVGRFDR at amino acids 430–436, similar to the RNA binding and unwinding motif (Q/H)RxGRxxR [55,59]. Additionally, an LRIR motif present in the MRV λ1 protein has some sequence similarity to those found in the vaccinia D1 subunit (LKPR) and West Nile Virus (WNV) NS3 (LRPR), both of which have NTPase/RTPase activities [60]. Similarly, an RDETGL motif found in MRV λ1 is also present in putative RTPases, such as vaccinia D1 subunit and WNV NS3. The N-terminal 187 amino acids of λ1, when expressed in bacteria, exhibited dsRNA binding activity as measured by southwestern analysis using radiolabeled DNA [61,62], and dsRNA affinity was lost when lysine residues within the predicted nucleotide binding motif (aa 9 and 11) were mutated to alanine. Higher affinity was suggested for ssRNA relative to dsRNA by gel retardation analysis, as would be predicted for a helicase. Bisaillon and Lemay then showed that λ1 expressed in yeast exhibits RTPase activities dependent on Mg^2+^ and Mn^2+^ divalent cations, and unwinds dsDNA and dsRNA radiolabeled tailed duplexes [63,64,65]. In these studies, HIS4-tagged λ1 was purified by affinity chromatography on zinc chelate affinity columns, and the absence of contaminating yeast enzymes was presumed by the observation that HIS4-tagged λ1 extracts retained activity despite pre-incubation at 42 °C, while the presence of contaminating enzymes from yeast expressing a negative control plasmid (HIS4-AOX1) would be thermosensitive at 42 °C. Though eloquent controls were included, it would be good to revisit these experiments now with λ1 that contains mutations in the putative enzymatic sites and demonstrate the loss of NTPase and RTPase activities.

**Figure 6:**
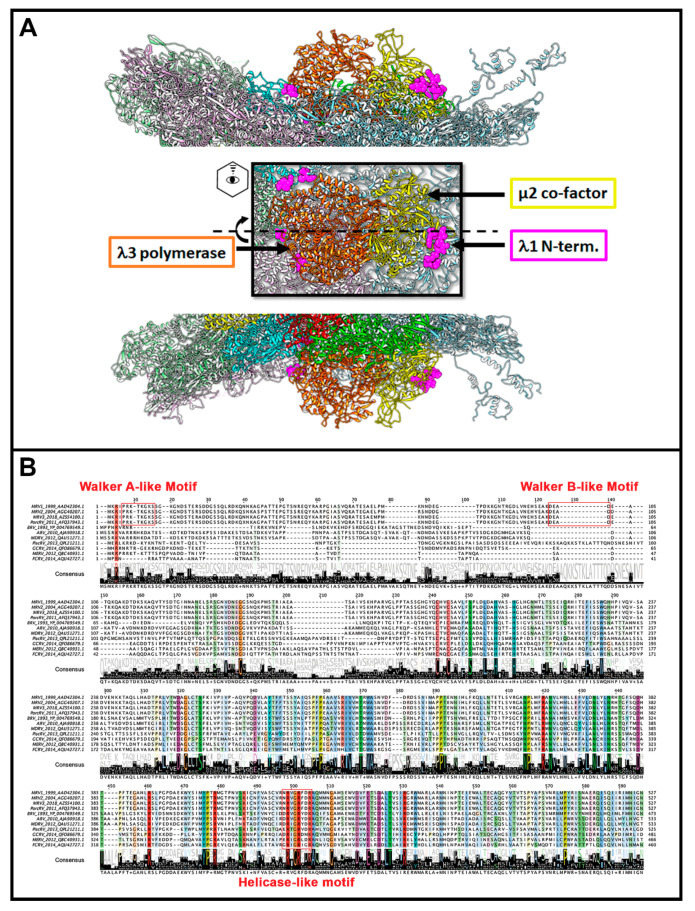
λ1 contact sites. (**A**) Structural depiction of a reovirus vertex. λ1 dimers are light-colored grey/pink/blue/green and derived from MRV core structure PDB 6M99 after superimposed onto the GCV VP3 λ1-homologue from PDB 1EJ6. The first resolvable amino acids at amino ends of the five vertex-proximal λ1 monomers are colored pink, representing the location of disordered N-termini proposed to have NTPase activity for MRV. The VP2 of GCRV (MRV λ3 RdRp homolog) and VP4 of GCRV (MRV μ2 homolog) are colored orange and yellow, respectively, and obtained from PDB 6M99; (**B**) Multiple sequence alignments of MRV λ1 homologs among *Orthoreoviruses* and *Aquareoviruses* acquired from ClustalOmega and visualized using Jalview 2.11.1.3. Accession numbers are provided in the figure. Conserved motifs (Walker A, Walker B, and helicase-like) are boxed in red. MRV, *Mammalian orthoreovirus*; PorcRV, *Porcine orthoreovirus*, BRV, *Baboon orthoreovirus*; ARV, *Avian orthoreovirus*; MDRV, *Muscovy duck reovirus*; GCRV, *Grass carp reovirus*; MERV, *Marbled eel reovirus*; FCRV, *Fall chinook aquareovirus*.

Although seemingly the role of λ1 as a phosphatase seems cut-and-dry, a mystery begins to unfold when considering the structure of reovirus cores and phylogenetic analysis between members of the *Spinareovirinae* subfamily (Figure 6). The core structures of *Orthoreovirus* (MRV) [2] and *Aquareovirus* (GCV) have been resolved by cryo-EM, and despite sequence divergence, the structures of core proteins between these genera are highly superimposable. Since only the structure of the GCV core includes the protein corresponding to MRV µ2 (called VP4), we generated a merged superimposed structure of MRV and GCV for analysis in our current review. The main body of the core is composed of λ1 dimers, with five orientated around the 5-fold vertex. For these five λ1 monomers, the first ~150 residues proposed to contain NTPase activities cannot be resolved for either MRV or GCV cores, suggesting either that they adopt a disordered structure or that they have asymmetrical orientation undecipherable by cryo-EM. Nevertheless, the approximate start location of λ1 termini can be inferred from the first ordered residues and place the termini at positions that encircle the GCV λ3 polymerase-homologue (called VP2) and µ2 homologue VP4 (Figure 6A, pink). One can therefore imagine that the remaining N-terminal residues may surround the RNA and rNTP entry and exit sites of the polymerase and participate in proposed functions such as dsRNA unwinding or rNTP hydrolysis. Here comes the twist: although the proposed Walker A and B motifs (Figure 6B) are well conserved among *Orthoreovirus,* the λ1 termini are highly divergent within and between genera, with most members completely devoid of those sites (Figure 6B). In fact, although we used the *Aquareovirus* structure to compose Figure 6, there is no sequence similarity between *Aquareovirus* and *Orthoreovirus* at their λ1 N-termini. Even members of *Orthoreovirus*, such as avian orthoreovirus (ARV) and baboon orthoreovirus (BRV), do not contain the proposed A and B ATPase sites. To help solve this mystery, it would be necessary to test if λ1 from genera other than *Orthoreovirus* also possess NTPase/RTPase, RNA/DNA binding, and helicase activities. As suggested earlier, it also seems necessary to mutate the MRV λ1 N-terminus to confirm if, indeed, the proposed ATPase-like sites are important for the RTPase and helicase activities discerned by λ1 expression in bacteria and yeast. Perhaps only λ1 of some *Spinareovirinae* participate in these roles, or perhaps different domains fulfill the same roles among distinct genera? Does divergence of the λ1 N-termini suggest a lack of evolutionary pressure to conserve sequence, structure and/or function, or rather does it suggest an evolutionary pressure for adaptation to specific cellular or environmental factors in distinct hosts?

**Figure 7:**
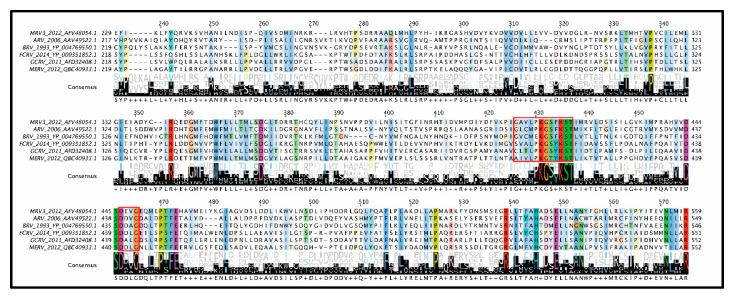
Conserved residues among μ2 homologs. Multiple sequence alignments for μ2 homologs among *Orthoreoviruses* and *Aquareoviruses* acquired using ClustalOmega and viewed using Jalview 2.11.1.3. Accession numbers are provided in the figure. Sequences of interest resembling the A (residues 426–437) and B (residues 462–465) motifs of ATPases are boxed in red. MRV3, *Mammalian orthoreovirus* serotype 3; ARV, *Avian orthoreovirus*; BRV, *Baboon orthoreovirus*; FCRV, *Fall chinook aquareovirus*; GCRV, *Grass carp reovirus*; MERV, *Marbled eel reovirus.*

Mu 2: As mentioned earlier, µ2 polymorphisms between MRV serotypes clearly correlate with changes in NTPase/RTPase activities, but whether this implies a direct or indirect role for µ2 in phosphohydrolase activity remains to be proven. When expressed in insect cells, µ2 did not exhibit ATPase activities, and radiolabeling and cross-linking of core proteins with oxidized [^32^P]ATP failed to show NTP binding by µ2 [56]. Conversely, µ2 bears some similarity to A and B motifs of ATPases, with T3D µ2 having GAVLPKGSFKS at positions 410–420 (depicted as residues 427–437 in Figure 7) and DEVG at positions 446–448 (depicted as residues 463–466 in Figure 7) [56]. Interestingly, the proposed NTPase domains found in MRV μ2 are highly conserved among *Orthoreovirus* and *Aquareovirus*, despite minimal amino acid sequence similarity with equivalent proteins (Figure 7). It should be noted that, at least for the *Orthoreoviruses*, µ2 helps facilitate the formation of viral factories (localized areas of virus replication and assembly); this secondary role likely also drives the structure and sequence evolution of the multifunctional µ2 protein. Strangely, when the conserved domains are mapped onto the *Aquareovirus* core structure (i.e., VP4 of GCV equivalent of µ2 of MRV), and their location considered relative to the polymerase (i.e., VP2 of GCV equivalent of λ3 of MRV), the conserved residues lie most proximal to the rNTP intake portal, second closest to the template entry portal, and facing opposite from the RNA exit portal (Figure 5C, black spheres). The possible contribution of µ2 to template unwinding is easy to imagine based on location, but where and when would RTPase activity on the nascent RNA strand occur if mediated by µ2?

**Figure 8:**
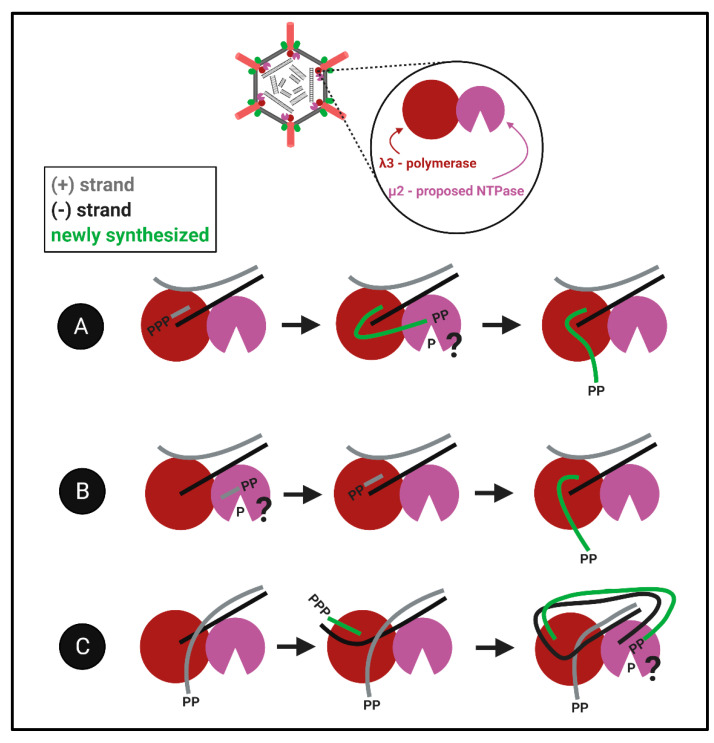
Proposed models for mRNA transcription mechanisms. Three proposed models for the RTP/NTPase accessing the 5′ terminus during transcription: (**A**) The nascent RNA exits the polymerase and undergoes 5′phosphohydrolysis before then returning to the polymerase for elongation followed by ejection; (**B**) The RTP/NTPase generates diphosphate primers that are then used by the polymerase for nascent strand generation; (**C**) Nascent RNA becomes the next (+) genomic RNA template that is then dephosphorylated after passing through the template exit portal. Figure generated using Biorender.com.

The proximity challenge for both λ1 and μ2: Although the evidence described above suggests that λ1 and μ2 could have RTPase activities, the largest bewilderment comes when considering the localization of these proteins relative to the well-defined portals of the RNA polymerase. Given that RNA synthesis occurs within the polymerase cavity, and nascent RNAs are then ejected through the λ2 channels, when would the RTP/NTPase activities of λ1 and/or μ2 have access to the 5′termini of the RNA? We imagine three models that may be compatible with findings (Figure 8): (Figure 8A) The nascent RNAs first exit into the core particle, undergo 5′phosphohydrolysis by λ1 and/or μ2, and then return into the polymerase for subsequent steps of capping, RNA elongation and ejection. (Figure 8B) The RTP/NTPase generates diphosphate primers, for example ppGpCp common to all genome segments, and these primers are used by the polymerase for nascent RNA synthesis. This possibility is congruent with discoveries of short oligonucleotides in reovirus particles [66]. (Figure 8C) The third more-complicated possibility we propose is that nascent RNAs become the next genomic (+)RNA templates and are dephosphorylated after passing through the template exit portal. In this model, the already diphosphate template would be ready for release and capping. The key to understanding the capping of reovirus RNAs will be to decipher between these models, and/or perhaps discover an alternative mechanism for hydrolysis of the 5′phosphate. Altogether, how members of the *Spinareovirinae* orchestrate the production of 5′diphosphate-bearing RNAs remains elusive, both in terms of which proteins and domains fulfill specific NTPase/RTPase functions, but also the spatiotemporal order of RNA polymerization versus 5′phosphohydrolysis steps. Many questions regarding the reovirus nucleotide phosphohydrolase are brought up in Box 4.

Box 4 **Unanswered Questions—Step 2: Nucleotide Phosphohydrolase.**

(1) Which viral proteins, μ2, λ1, or other, directly fulfil the following functions?
  - NTPase-dependent template unwinding
  - RTPase step of RNA capping
  - Genomic RNA selection and packaging
(2) Which domains of μ2, λ1, or other proteins fulfil these functions, and what is the direct evidence to support domain activity?
(3) Do all vertices behave the same, or can the same protein at a different vertex have a distinct NTPase/RTPase specificity?
(4) Do the same (or different) proteins and domains fulfil the same functions among all Spinareovirinae, or have the genera diverged in their protein functions?
(5) Given that the proposed NTPase and RTPase lie on the outside of the polymerase…
  - Do they access the 5’ end of the nascent RNAs?
  - Does the nascent RNA briefly exit the polymerase into the core for phosphohydrolysis?
  - Does the RTPase instead generate ppGpCp primers that are used by the polymerase? Or is there another mechanism?
(6) Given several proposed NTPase activities…
  - How does the virus prevent depletion of NTPs for RNA synthesis?
  - Do NTPase activities play additional roles, such as to deplete NTPs and regulate core transcription?

**Step 3: Transfer of inverted GMP to 5′ end of RNA via guanylyltransferase.**

**Figure 9:**
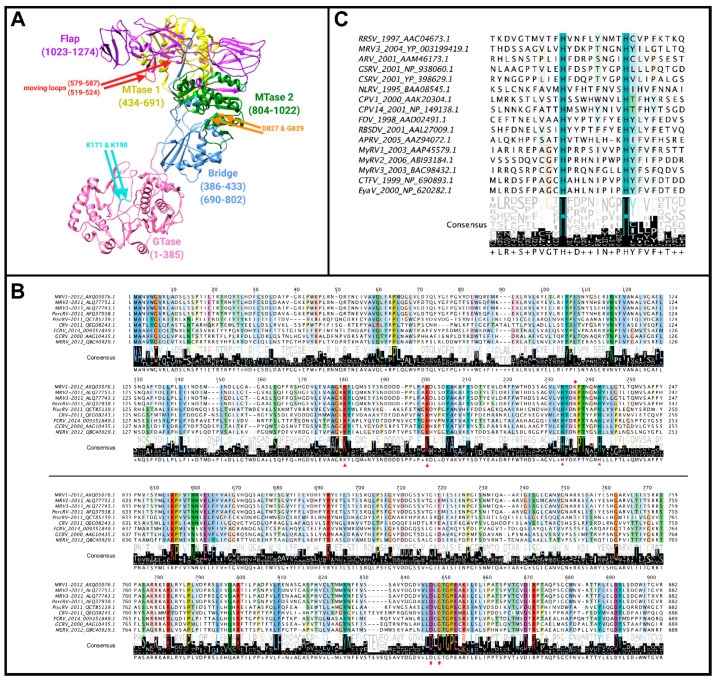
*Spinareovirinae* turret protein structure (MRV) and sequence alignments. (**A**) The λ2 monomer (PDB 1EJ6) with highlighted domains labelled. The moving loops (red) within the MTase 1 domain are highlighted using arrows. Lysine residues at position 171 and 190 are also highlighted using arrows (cyan) within the active site of the GTase domain. Figure generated using Biorender.com; (**B**) Multiple sequence alignment of λ2 homologs among *Orthoreoviruses* and *Aquareoviruses* acquired using ClustalOmega and viewed using Jalview 2.11.1.3. Accession numbers are provided in the figure. Conserved lysine residues 181 and 201, corresponding to MRV λ2 K171 and K190, are denoted by arrowheads. Conserved histidines 234 and 243, corresponding to MRV λ2 H223 and H232, are denoted with stars. Lysine 226 found in certain *Orthoreoviruses* is denoted with a diamond. Conserved aspartic acid 846 and glycine 848, corresponding to MRV λ2 D827 and G829, are denoted with arrows; (**C**) Multiple sequence alignment of λ2 homologs among all *Spinareovirinae* genera acquired using ClustalOmega and viewed using Jalview 2.11.1.3. MRV, *Mammalian orthoreovirus*; PorcRV, *Porcine orthoreovirus*, PiscRV, *Piscine orthoreovirus*; CRV, *Carp reovirus*; FCRV, *Fall chinook aquareovirus*; GCRV, *Grass carp reovirus*; MERV, *Marbled eel reovirus*; RRSV, *Rice ragged stunt virus*; GSRV, *Golden shiner reovirus*; CSRV, *Chum salmon reovirus*; NLRV, *Nilaparvata lugens reovirus*; CPV, *Cypovirus*; FDV, *Fiji disease virus*; RBSDV, *Rice black-streaked dwarf virus*; MyRV, *Mycoreovirus*; CTFV, *Colorado tick fever virus*; EyaV, *Eyach virus.*

Once a 5′-diphopshate end has been generated on the nascent (+)RNA molecule by RTPase, a backwards guanosine monophosphate (GMP) group from GTP can be added through guanylyltransferase (GTase) activity, as shown in Figure 3, step #3. In the case of MRV, the guanylyltransferase enzyme was identified first by Cleveland et al. [67]. Using [^32^P] to label the covalent enzyme-guanylate intermediate, the λ2 turret protein was identified as the major capping enzyme. For *Spinareovirinae,* each of the 12 vertices consists of a pentameric channel formed by λ2 proteins. The discovery of guanylyltransferase activity in λ2 suggested that viral (+)RNAs are capped during their extrusion from the λ2 channels. In 1987, the λ2 protein was sequenced [68], allowing the precise region that bound [^32^P] GMP to be determined. The region was proposed to be residues 213–269, with GMP attaching to lysine 226. The structure of MRV λ2, including the location of enzymatic motifs, is highlighted in Figure 9A.

To elucidate the precise enzymatic activities of λ2, Mao et al. cloned the L2 sequence into vaccinia virus and subsequently isolated it from infected cell cultures [69]. They found that GTP did not label λ2 if [^32^P] was in the γ-position, nor was λ2 labelled by ATP, CTP, or UTP. This suggested that λ2 did not possess a generic NTP-binding activity, but instead must be specific. Incubating λ2 with unlabelled GTP and [^32^P] PP_i_ resulted in the formation of [^32^P] GTP, demonstrating that λ2 catalyzed this formation. Taking these studies together, the GTase activity of λ2 was apparent. Following this discovery, Luongo et al. determined it was precisely the N-terminal 42 kDa fragment of λ2 that mediates the GTase activity, as this fragment retained both linkage to GMP using a GTP substrate and the transfer of GMP to an acceptor molecule (GDP or GTP). In their 2000 publication, Luongo et al. found that the λ2-GMP covalent intermediate was not actually mediated by lysine 226 as suggested earlier; mutation of this residue to alanine did not completely abolish GMP association [70]. Mutating lysines 171 and 190 to alanine caused greater loss of GMP association, thus these residues were presumed to be the site of GMP linkage [70,71,72]. The authors suggest that their mutations are unlikely to affect λ2 structure, and hence GMP linkage directly, as their mutant proteins still undergo similar proteolysis to wild-type. This is further supported by the conservation of lysine at position 190 among λ2 proteins of MRV serotypes 1, 2, and 3, which share ~86–92% sequence identity in λ2, whereas the previously mentioned lysine 226 residue appears to only be conserved among *Orthoreovirus* members (Figure 9B) [70]. Nevertheless, a direct assay implicating lysine 190 covalent attachment to GMP would be conclusively convincing.

In *Aquareovirus*, the protein homologous to MRV λ2, called VP1, was found to possess GTase activity after it was isolated from a Baculovirus expression system and incubated with [^32^P] GTP. Chymotrypsin digestion of VP1 in the presence of labelled GTP produced a 100 kDa unlabelled fragment, and a 42 kDa labelled fragment suggested to contain GTase activity. Despite only 28% sequence homology between GCV VP1 and GCV λ2, VP1 also contained lysine residues at positions 176 and 196 in the 42 kDa fragment, similar to GTase-associated lysines 171 and 190 in MRV λ2 [73] (Figure 9B). Crystal structure alignments with VP1 and λ2 reveal the VP1 turret channels to be smaller, and the GTase domains to contain only scattered conservation on the bottom surface that interacts with the MRV homolog λ1, known as VP3. In contrast, along the top of the GTase domain, a continuous segment including the aforementioned lysines 171 and 190 is well conserved between VP1 and λ2. Furthermore, the *Cypovirus* protein VP3 also mediates GTase activity, and like VP1, shares no significant sequence homology to λ2 despite having similar topology [60]. Accordingly, homologs of MRV λ2 from other *Spinareovirinae* genera may exhibit structural and functional conservation despite lacking sequence similarity.

Eukaryotes and DNA viruses are known to contain conserved Kx[D/N]G motifs within their GTases; however, *Spinareovirinae* lacks this sequence [71]. Yet, based on sequence comparisons, a Kx[V/L/I]S motif is present in all known and proposed GTases of *Spinareovirinae*. Using site-directed mutagenesis to change lysine to alanine, Luongo et al. found that only lysine 190 of the ^190^KDLS sequence is necessary for enzymatic activity [71]. Upon investigating the effects of the other three residues (D191, L192, and S193), they found that D191A mutation also demonstrated reduced enzymatic activity; however, mutations in L192 and S193 had no significant difference [71]. This suggests that the *Spinareovirinae* ^190^KDLS sequence may not be functionally equivalent to the eukaryotic and DNA virus GTase motif, Kx[D/N]G.

Historically, early studies on the MRV λ2′s GTase activity focused on active site lysine residues and the conserved KDLS sequence, but later studies revealed the potential role of histidine residues (Figure 9C). In their 2003 study, Qiu and Luongo showed that *Aquareovirus* VP1 functions optimally at pH 5.0, leading the authors to believe that histidine protonation may be affecting the activity [73]. They proposed two hypotheses for how protonation of histidine residues could mediate GTase activity: (1) two solvent exposed (+) charges may help to neutralize the negative phosphate residues and thus increase affinity for the enzyme, and (2) protonation could cause the loop at position 223–232 to move, resulting in movement across adjacent residues and enhancing GTP binding via conserved lysine residues [73]. Further supporting their findings, site-directed mutagenesis of two histidine residues conserved between MRV, ARV, and GCV GTases revealed that they are essential for enzymatic activity. In the MRV GTase domains, histidine residues at positions 223 and 232 were conserved, and mutating them to alanine abolished GTase activity despite no apparent disruptions to protein folding [73]. Additionally, other homologous proteins to λ2 in *Spinareovirinae* genera (P2 of *Oryzavirus*, VP3 of *Mycoreovirus*, VP3 of *Fijivirus*, and VP2 of *Coltivirus*) (Figure 9C) were also found to contain critical histidine residues in their GTase domains [4,74]. Interestingly, site-directed mutagenesis studies with *Mycoreovirus* VP3 found that histidine residues at positions 233 and 242 played critical roles for GTase activity, while lysines 172 and 202 did not [74]. This further supported the notion that an Hx(8)H motif makes up the GTase active site. Supyani et al. [74] stated that the Hx(8)H motif is conserved among all members of *Spinareovirinae*, suggesting this motif is enzymatically significant.. However, it remains to be determined if the conserved histidine residues are responsible for GTase activity across all generas of *Spinareovirinae*. Questions pertaining to the reovirus GTase are highlighted in Box 5.

Box 5 **Unanswered Questions—Step 3: Guanylyltransferase.**

(1) Are conserved lysine or histidine residues mediating GTase activity… Or both?
  - Are these residues responsible for activity among all *Spinareovirinae* members, or does it differ?
  - Are the aforementioned residues directly or indirectly involved in enzymatic activity?
(2) Do findings with monomeric proteins (λ2) translate to proteins in complexes as well?
(3) If the mutational studies are repeated using current reverse genetic systems, will findings be recapitulated?
(4) Do the turret proteins of all *Spinareovirinae* members possess GTase activity? Or could this action be mediated by another protein?

**Steps 4 and 5: N7 methylation (and possibly more) for cap production via methyl transferases.**

The final steps of the capping reaction consist of methylation reactions as shown in Figure 3 steps #4–6. First, the guanosine cap structure is methylated at the 7-N position by a guanosine-7-N-methyltransferase (MTase 1). Second, the first nucleotide of the RNA chain is methylated by a guanosine-2′-*O*-methyltransferase (MTase 2). As discussed earlier in this review, it is uncertain if reovirus RNAs can also have cap2 structures, but should cap2 structures exist, then the second nucleotide of the RNA chain would also become methylated by a guanosine-2′-*O*-methyltransferase (MTase 3). For MRV, MTase1 and MTase 2 methylation reactions have been associated with the λ2 protein, which has been suggested to catalyze methyl group transfer in addition to its GTase activity. However, other members of *Spinareovirinae (Coltivirus, Oryzavirus)* are proposed to encode their GTase and methyltransferase (MTase) activities within separate proteins. Initially, unpublished findings suggested that λ2 is labelled in the presence of 8-azid-adenosyl[^35^S]methionine, as described in [68]. Luongo et al. (1998) then found that λ2 was the only MRV protein bound to SAM after UV-crosslinking [75]. Moreover, the SAM-binding domain was within the central region of λ2 (amino acids 792–1100), which includes a region of similarity to SAM-binding pockets of other MTases such as flavivirus NS5 proteins and yeast CoQ3 [75,76]. Alanine mutations at positions D827 and G829 of λ2 reduced the SAM-binding capacity of a baculovirus-produced λ2. Being central to the two MTase activities, the authors hypothesized that reovirus utilizes a single SAM-binding pocket for both MTase 1 and MTase 2 reactions. However, the authors recognized that a second less-avid SAM-binding pocket could also exist but remain undetectable by their assays. Nevertheless, the idea of a single SAM-binding domain does lend to an interesting hypothesis; that SAM-binding domains of λ2 monomers donate SAM to MTases of partner λ2 monomers in the pentamer.

That λ2 might need to work as a pentamer for methylation would help explain why purified λ2 monomers retain GTase activity but lack MTase activities [69]. In λ2 pentamers, the GTase site of one monomer is closer to the MTase 1 site in the clockwise neighbour than to its own MTase domains, exposing the possibility that a cooperative mechanism is at play [77]. However, alternatively, the requirement for multimers might simply reflect a conformational difference required for MTase activity, rather than cooperation between monomers for activity.

Studies with *Cypovirus* have revealed greater insights into possible cooperativity mediating MTase activities. Using NTPase assays and comparisons of cryo-EM images of cypovirus (CPV) with bound ligands, Yu et al. determined that the CPV turret protein VP3 (λ2 homologue) also contains an ATPase site [78]. To our knowledge, an ATPase site in MRV λ2 has yet to be described. Yu et al. also determined that in CPV, SAM binds to MTase 2, inducing a conformational change in the viral capsid that activates the ATPase. ATP binding/hydrolysis induces an enlarged capsid, supposedly for efficient mRNA synthesis, an open GTase domain, and an open MTase 1 domain for subsequent SAM binding and methyl transfer. Another unique feature of CPV VP3 is that in addition to the GTase, bridge, and two MTase domains, VP3 has an extra “brace domain” between the GTase and MTase 1 domains [60]. The “brace domain” of CPV VP3 is proposed by Cheng et al. to guide nascent mRNAs from the GTase of one VP3 copy, to the MTase 1 domain of the neighboring VP3, to the MTase 2 domain of the next neighbor. Although CPV displays a different turret micro-structure than MRV, the enzymatic sequence of events may remain the same. Nevertheless, whether monomers function cooperatively in the pentameric turret structure to fulfil methylation reactions remains to be empirically established among the *Spinareoviridae* members.

Using a bioinformatics approach of structure and sequence similarities, Bujnicki and Rychlewski (2001) proposed a new assignment of MTase domains [77]. They noted that the MTase 1 domain, described by Reinish et al. (2000) [2], bears the highest resemblance to 2′O-MTases: the *Escherichia coli* 23S ribosomal RNA MTase and the bonafide cap1 MTase of vaccinia virus. As such, Bujnicki and Rychlewski proposed that the designations be swapped so that the MTase2 domain mediates 2′O-methylation of the first mRNA nucleotides while the MTase1 domain mediates the N-methylation of the guanosine cap. Although the argument provided by Bujnicki and Rychlewski is convincing, it is surprising given the spatial order of these domains (Figure 9A, yellow versus green domains). Specifically, the suggestion by Bujnicki and Rychlewski would place the 2′O-MTase closer to the GTase than the 7′N MTase in a λ2 monomer, so is it possible that 2′O-methylation of the first nucleotide comes before the 7′N-methylation of the guanosine cap? More likely is that, similar to proposed for CPV VP3, the RNA is passed between neighboring turret proteins to reach MTase domains in the appropriate order. Unanswered questions regarding the reovirus MTase are highlighted in Box 6.

Box 6 **Unanswered Questions—Step 4: MTase.**

(1) Does λ2 contain one or two SAM-binding pockets? If only one, then…
  - Does the binding domain in one λ2 monomer function cooperatively with another?
  - Do only λ2 multimers exhibit methyltransferase activity? How many monomers facilitate the reaction?
  - Does the methyltransferase activity of λ2 require a larger structure, or is it part of a currently unknown process?
(2) Is there a direct assay that could implicate the appropriate MTase reaction with their respective domains?
  - Which domain adds the cap0, cap1, and cap2 structures? Is it consistent with the current proposed models/reaction mechanisms?

Host cytoplasmic RNA capping. Although we have discussed reovirus RNA capping as a viral process, it is important to consider the fact that mammalian cells also have cytoplasmic 5′ capping enzymes. For a detailed description of mammalian cytoplasmic RNA capping, we recommend visiting a recent review [79] as well as the discovery of the cytoplasmic capping complex [80] to fully appreciate the subject. It was previously believed that the loss of the 5′ RNA cap leads to RNA degradation [79]. However, Otsuka et al. discovered a cytoplasmic enzyme complex containing a bifunctional RNA guanylyltransferase-5′ phosphatase (RNGTT), a monophosphate kinase, and guanine-N7 methyltransferase (RNMT) that, together, were capable of converting 5′ monophosphates to a GpppN 5′ terminus [80]. Despite the fact that reovirus encodes its own capping machinery, as described above, it is possible that host cytoplasmic capping enzymes also contribute to the cap status of reovirus RNAs, for example, by generating cap2 structures. In the future, it would be interesting to decipher the cap structures on reovirus RNAs in cells depleted of cellular cytoplasmic capping enzymes.

## 5. Closing Remarks

Despite the discovery of mRNA caps originating from studies on *Cypovirus*, many questions remain regarding the exact structure, function, and process of mRNA capping in the context of *Spinareovirinae*. The work of many researchers over the past four decades has revealed several structures of caps found on viral (+)RNA over the course of an infection, namely uncapped, cap0, cap1 and cap2 structures (Figure 4C); however, which of these is essential for virus replication, or if different structures dictate the fate of nascent mRNA, is still largely unknown. Several studies have established that capped RNA contributes to viral RNA translation. However, whether the cap(s) play(s) a role outside of translation, such as virion assembly and RNA transcription, also remains a subject for future investigation. Furthermore, while the precise role(s) of viral proteins in the RNA capping process is (are) becoming unravelled, especially for MRV, there is still ambiguity when it comes to identifying key enzymatic domains/residues. For instance, whether the MTase activity of MRV λ2, and respective homologs, required complex formation is still a mystery to be solved. Which proteins facilitate the RTPase step of capping remains enigmatic. Finally, translating findings from one genus of *Spinareovirinae* to another is also a challenge researchers have faced due to the overall low conservation in some protein sequences (not to mention the unfortunate disparity in the nomenclature of functionally homologous proteins between *Spinareovirinae* members). Future research on *Spinareovirinae* RNA capping will provide experimental evidence that directly supports or refutes specific concepts discussed in this review, and ideally will produce a coherent model of this fundamental process.

## References

[1] Andrew M.Q., King M.J.A., Carstens E.B., Lefkowitz E.J. (2012). Virus Taxonomy.

[2] Reinisch K.M., Nibert M.L., Harrison S.C. (2000). Structure of the reovirus core at 3.6?Å resolution. Nature.

[3] Chandran K., Walker S.B., Chen Y.A., Contreras C.M., Schiff L.A., Baker T.S., Nibert M.L. (1999). In Vitro Recoating of Reovirus Cores with Baculovirus-Expressed Outer-Capsid Proteins 1 and 3. J. Virol..

[4] Miyazaki N., Uehara-Ichiki T., Xing L., Bergman L., Higashiura A., Nakagawa A., Omura T., Cheng R.H. (2008). Structural Evolution of Reoviridae Revealed by Oryzavirus in Acquiring the Second Capsid Shell. J. Virol..

[5] Li X., Fang Q. (2013). High-resolution 3D structures reveal the biological functions of reoviruses. Virol. Sin..

[6] Dryden K.A., Wang G., Yeager M., Nibert M.L., Coombs K.M., Furlong D.B., Fields B.N., Baker T.S. (1993). Early Steps in Reovirus Infection Are Associated with Dramatic Changes in Supramolecular Structure and Protein Conformation: Analysis of Virions and Subviral Particles by Cryoelectron Microscopy and Image Reconstruction. J. Cell Biol..

[7] Snyder A.J., Wang J.C.-Y., Danthi P. (2018). Components of the reovirus capsid differentially contribute to stability. J. Virol..

[8] Kim J., Tao Y., Reinisch K.M., Harrison S.C., Nibert M.L. (2004). Orthoreovirus and Aquareovirus core proteins: Conserved enzymatic surfaces, but not protein-protein interfaces. Virus Res..

[9] Roth A.N., Aravamudhan P.A. (2020). Ins and outs of reovirus: Vesicular trafficking in viral entry and egress. Trends Microbiol..

[10] Barton E.S., Connolly J.L., Forrest J.C., Chappell J.D., Dermody T.S. (2001). Utilization of sialic acid as a coreceptor enhances reovirus attachment by multistep adhesion strengthening. J. Biol. Chem..

[11] Schulz W.L., Haj A.K., Schiff L.A. (2012). Reovirus uses multiple endocytic pathways for cell entry. J. Virol..

[12] Mainou B.A., Dermody T.S. (2012). Transport to late endosomes is required for efficient reovirus infection. J. Virol..

[13] Amerongen H.M., Wilson G.A.R., Fields B.N., Neutral M.R. (1994). Proteolytic Processing of Reovirus Is Required for Adherence to Intestinal M Cells. J. Virol..

[14] Shmulevitz M., Epand R.F., Epand R.M., Duncan R. (2004). Structural and functional properties of an unusual internal fusion peptide in a nonenveloped virus membrane fusion protein. J. Virol..

[15] Tenorio R., de Castro I.F., Knowlton J.J., Zamora P.F., Sutherland D.M., Risco C., Dermody T.S. (2019). Function, architecture, and biogenesis of reovirus replication neoorganelles. Viruses.

[16] Furuichi Y., Shatkin A.J. (1976). Differential synthesis of blocked and unblocked 5′-termini in reovirus mRNA: Effect of pyrophosphate and pyrophosphatase. Proc. Natl. Acad. Sci. USA.

[17] Furuichi Y., Muthukrishnan S., Tomasz J., Shatkin A.J. (1976). Mechanism of formation of reovirus mRNA 5′-terminal blocked and methylated sequence, m7GpppGmpC. J. Biol. Chem..

[18] Kozak M., Shatkin A.J. (1976). Characterization of ribosome-protected fragments from reovirus messenger RNA. J. Biol. Chem..

[19] Shatkin A.J., Both G.W. (1976). Reovirus mRNA: Transcription and translation. Cell.

[20] Furuichi Y., Muthukrishnan S., Tomasz J., Shatkin A.J. (1976). Caps in eukaryotic mRNAs: Mechanism of formation of reovirus mRNA 5′-terminal m7GpppGm-C. Prog. Nucleic Acid Res. Mol. Biol..

[21] Both G.W., Furuichi Y., Muthukrishnan S., Shatkin A.J. (1975). Ribosome binding to reovirus mRNA in protein synthesis requires 5′ terminal 7-methylguanosine. Cell.

[22] Both G.W., Lavi S., Shatkin A.J. (1975). Synthesis of all the gene products of the reovirus genome in vivo and in vitro. Cell.

[23] Chow N.L., Shatkin A.J. (1975). Blocked and unblocked 5′ termini in reovirus genome RNA. J. Virol..

[24] Furuichi Y., Morgan M., Muthukrishnan S., Shatkin A.J. (1975). Reovirus messenger RNA contains a methylated, blocked 5′-terminal structure: M-7G(5′)ppp(5′)G-MpCp. Proc. Natl. Acad. Sci. USA.

[25] Furuichi Y., Muthukrishnan S., Shatkin A.J. (1975). 5′-Terminal m-7G(5′)ppp(5′)G-m-p in vivo: Identification in reovirus genome RNA. Proc. Natl. Acad. Sci. USA.

[26] Muthukrishnan S., Shatkin A.J. (1975). Reovirus genome RNA segments: Resistance to S-1 nuclease. Virology.

[27] Sen G.C., Lebleu B., Brown G.E., Rebello M.A., Furuichi Y., Morgan M., Shatkin A.J., Lengyel P. (1975). Inhibition of reovirus messenger RNA methylation in extracts of interferon-treated Ehrlich ascites tumor cells. Biochem. Biophys. Res. Commun..

[28] Miura K., Watanabe K., Sugiura M., Shatkin A.J. (1974). The 5′-terminal nucleotide sequences of the double-stranded RNA of human reovirus. Proc. Natl. Acad. Sci. USA.

[29] Shatkin A.J. (1974). Methylated messenger RNA synthesis in vitro by purified reovirus. Proc. Natl. Acad. Sci. USA.

[30] Furuichi Y. (1974). "Methylation-coupled" transcription by virus-associated transcriptase of cytoplasmic polyhedrosis virus containing double-stranded RNA. Nucleic Acids Res..

[31] Rhodes D.P., Moyer S.A., Banerjee A.K. (1974). In vitro synthesis of methylated messenger RNA by the virion-associated RNA polymerase of vesicular stomatitis virus. Cell.

[32] Wei C.M., Moss B. (1974). Methylation of newly synthesized viral messenger RNA by an enzyme in vaccinia virus. Proc. Natl. Acad. Sci. USA.

[33] Banerjee A.K., Ward R., Shatkin A.J. (1971). Cytosine at the 3′-termini of reovirus genome and in vitro mRNA. Nat. New Biol..

[34] Borsa J., Grover J., Chapman J.D. (1970). Presence of nucleoside triphosphate phosphohydrolase activity in purified virions of reovirus. J. Virol..

[35] Desrosiers R.C., Sen G.C., Lengyel P. (1976). Difference in 5′ terminal structure between the mRNA and the double-stranded virion RNA of reovirus. Biochem. Biophys. Res. Commun..

[36] Desrosiers R.C., Lengyel P. (1979). Impairment of reovirus mRNA ‘cap’ methylation in interferon-treated mouse L929 cells. Biochim. Biophys. Acta.

[37] Sen G.C., Shaila S., Lebleu B., Brown G.E., Desrosiers R.C., Lengyel P. (1977). Impairment of reovirus mRNA methylation in extracts of interferon-treated Ehrilich ascites tumor cells: Further characteristics of the phenomenon. J. Virol..

[38] Skup D., Millward S. (1980). mRNA capping enzymes are masked in reovirus progeny subviral particles. J. Virol..

[39] Zarbl H., Skup D., Millward S. (1980). Reovirus progeny subviral particles synthesize uncapped mRNA. J Virol..

[40] Skup D., Zarbl H., Millward S. (1981). Regulation of translation in L-cells infected with reovirus. J. Mol. Biol..

[41] Mohamed A., Clements D.R., Gujar S.A., Lee P.W., Smiley J.R., Shmulevitz M. (2019). Single amino acid differences between closely related reovirus T3D lab strains alter oncolytic potency in vitro and in vivo. J. Virol..

[42] Mohamed A., Konda P., Eaton H.E., Gujar S., Smiley J.R., Shmulevitz M. (2020). Closely related reovirus lab strains induce opposite expression of rig-i/ifn-dependent versus -independent host genes, via mechanisms of slow replication versus polymorphisms in dsrna binding σ3 respectively. PLoS Pathogens.

[43] Mohamed A., Smiley J.R., Shmulevitz M. (2019). Polymorphisms in the most oncolytic reovirus strain confer enhanced cell attachment, transcription, and single-step replication kinetics. J. Virol..

[44] Reeve A.E., Shatkin A.J., Huang R.C. (1982). Guanosine 5′-O-(3-thiotriphosphate) inhibits capping of reovirus mRNA. J. Biol. Chem..

[45] Kozak M., Shatkin A.J. (1978). Identification of features in 5′ terminal fragments from reovirus mRNA which are important for ribosome binding. Cell.

[46] Eaton H.E., Kobayashi T., Dermody T.S., Johnston R.N., Jais P.H., Shmulevitz M. (2017). African swine fever virus NP868R capping enzyme promotes reovirus rescue during reverse genetics by promoting reovirus protein expression, virion assembly, and RNA incorporation into infectious virions. J. Virol..

[47] Shmulevitz M., Pan L.Z., Garant K., Pan D., Lee P.W. (2010). Oncogenic Ras promotes reovirus spread by suppressing IFN-beta production through negative regulation of RIG-I signaling. Cancer Res..

[48] Goubau D., Schlee M., Deddouche S., Pruijssers A.J., Zillinger T., Goldeck M., Schuberth C., Van der Veen A.G., Fujimura T., Rehwinkel J. (2014). Antiviral immunity via RIG-I-mediated recognition of RNA bearing 5′-diphosphates. Nature.

[49] Starnes M.C., Joklik W.K. (1993). Reovirus protein λ3 is a poly(c)-dependent poly(g) polymerase. Virology.

[50] Kaelber J.T., Jiang W., Weaver S.C., Auguste A.J., Chiu W. (2020). Arrangement of the polymerase complexes inside a nine-segmented dsRNA virus. Structure.

[51] Tao Y., Farsetta D.L., Nibert M.L., Harrison S.C. (2002). RNA synthesis in a cage—Structural studies of reovirus polymerase λ3. Cell.

[52] Yamakawa M., Furuichi Y., Nakashima K., LaFiandra A.J., Shatkin A.J. (1981). Excess synthesis of viral mRNA 5′-terminal oligonucleotides by reovirus transcriptase. J. Biol. Chem..

[53] Farsetta D.L., Chandran K., Nibert M.L. (2000). Transcriptional activities of reovirus RNA polymerase in recoated cores: Initiation and elongation are regulated by separate mechanisms. J. Biol. Chem..

[54] Kapuler A.M., Mendelsohn N., Klett H., Acs G. (1970). Four base-specific nucleoside 5′-triphosphatases in the subviral core of reovirus. Nature.

[55] Noble S., Nibert M.L. (1997). Characterization of an ATPase activity in reovirus cores and its genetic association with core-shell protein lambda1. J. Virol..

[56] Noble S., Nibert M.L. (1997). Core protein mu2 is a second determinant of nucleoside triphosphatase activities by reovirus cores. J. Virol..

[57] Yin P., Cheang M., Coombs K.M. (1996). The M1 gene is associated with differences in the temperature optimum of the transcriptase activity in reovirus core particles. J. Virol..

[58] Coombs K.M. (1996). Identification and characterization of a double-stranded RNA- reovirus temperature-sensitive mutant defective in minor core protein mu2. J. Virol..

[59] Bartlett J.A., Joklik W.K. (1988). The sequence of the reovirus serotype 3 L3 genome segment which encodes the major core protein lambda 1. Virology.

[60] Cheng L., Sun J., Zhang K., Mou Z., Huang X., Ji G., Sun F., Zhang J., Zhu P. (2011). Atomic model of a cypovirus built from cryo-EM structure provides insight into the mechanism of mRNA capping. Proc. Natl. Acad. Sci. USA.

[61] Lemay G., Danis C. (1994). Reovirus λ1 protein: Affinity for double-stranded nucleic acids by a small amino-terminal region of the protein independent from the Zinc finger motif. J. Gen. Virol..

[62] Bisaillon M., Lemay G. (1997). Molecular dissection of the reovirus lambda1 protein nucleic acids binding site. Virus Res..

[63] Bisaillon M., Bergeron J., Lemay G. (1997). Characterization of the nucleoside triphosphate phosphohydrolase and helicase activities of the reovirus lambda1 protein. J. Biol. Chem..

[64] Bisaillon M., Lemay G. (1997). Characterization of the reovirus lambda1 protein RNA 5′-triphosphatase activity. J. Biol. Chem..

[65] Bisaillon M., Lemay G. (1997). Viral and cellular enzymes involved in synthesis of mRNA cap structure. Virology.

[66] Bellamy A.R., Hole L.V. (1970). Single-stranded oligonucleotides from reovirus type III. Virology.

[67] Cleveland D.R., Zarbl H., Millward S. (1986). Reovirus guanylyltransferase is L2 gene product lambda 2. J. Virol..

[68] Seliger L.S., Zheng K., Shatkin A.J. (1987). Complete nucleotide sequence of reovirus L2 gene and deduced amino acid sequence of viral mRNA guanylyltransferase. J. Biol. Chem..

[69] Mao Z.X., Joklik W.K. (1991). Isolation and enzymatic characterization of protein lambda 2, the reovirus guanylyltransferase. Virology.

[70] Breun L.A., Broering T.J., McCutcheon A.M., Harrison S.J., Luongo C.L., Nibert M.L. (2001). Mammalian reovirus L2 gene and lambda2 core spike protein sequences and whole-genome comparisons of reoviruses type 1 Lang, type 2 Jones, and type 3 dearing. Virology.

[71] Luongo C.L. (2002). Mutational analysis of a mammalian reovirus mRNA capping enzyme. Biochem. Biophys. Res. Commun..

[72] Luongo C.L., Reinisch K.M., Harrison S.C., Nibert M.L. (2000). Identification of the guanylyltransferase region and active site in reovirus mRNA capping protein λ2. J. Biol. Chem..

[73] Qiu T., Luongo C.L. (2003). Identification of two histidines necessary for reovirus mRNA guanylyltransferase activity. Virology.

[74] Supyani S., Hillman B.I., Suzuki N. (2007). Baculovirus expression of the 11 mycoreovirus-1 genome segments and identification of the guanylyltransferase-encoding segment. J. Gen. Virol..

[75] Luongo C.L., Contreras C.M., Farsetta D.L., Nibert M.L. (1998). Binding site for S-adenosyl-L-methionine in a central region of mammalian reovirus lambda2 protein. Evidence for activities in mRNA cap methylation. J. Biol. Chem..

[76] Koonint E.V. (1993). Computer-Assisted Identification of a Putative Methyltransferase Domain in NS5 Protein of Flaviviruses and 22 Protein of Reovirus. J. Gen. Virol..

[77] Bujnicki J.M., Rychlewski L. (2001). Reassignment of specificities of two cap methyltransferase domains in the reovirus lambda 2 protein. Genome Biol..

[78] Yu X., Jiang J., Sun J., Zhou Z.H. (2015). A putative ATPase mediates RNA transcription and capping in a dsRNA virus. eLife.

[79] Trotman J.B., Schoenberg D.R. (2019). A recap of RNA recapping. WIREs RNA.

[80] Otsuka Y., Kedersha N.L., Schoenberg D.R. (2009). Identification of a cytoplasmic complex that adds a cap onto 5′-Monophosphate RNA. Mol. Cell. Biol..

